# Effects of Dentine Pretreatment Solutions Containing Flavonoids on the Resin Polymer-Dentine Interface Created Using a Modern Universal Adhesive

**DOI:** 10.3390/polym13071145

**Published:** 2021-04-02

**Authors:** Andrés Dávila-Sánchez, Mario Felipe Gutierrez, Jorge Pailover Bermudez, Luján Méndez-Bauer, Camilo Pulido, Fagner Kiratzc, Luisa Fernanda Alegria-Acevedo, Paulo Vitor Farago, Alessandro Dourado Loguercio, Salvatore Sauro, Cesar Augusto Galvão Arrais

**Affiliations:** 1 Universidad San Francisco de Quito USFQ, Departmento de Odontología Restauradora y Materiales Dentales, Escuela de Odontología, Pampite y Diego de Robles, Quito 170901, Ecuador; cadavilas@usfq.edu.ec (A.D.-S.); cpulido@usfq.edu.ec (C.P.); 2 Universidad de los Andes, Chile, Facultad de Odontología, Monseñor Álvaro del Portillo 12455, Santiago 7550000, Chile; gutierrezreyes.felipe@gmail.com; 3 Universidad Finis Terrae, Chile, Facultad de Odontología, Av. Pedro de Valdivia 1509, Santiago 7501015, Chile; 4 Department of Restorative Dentistry, State University of Ponta Grossa, School of Dentistry, Ponta Grossa, Paraná 840030-900, Brazil; pailover14@hotmail.com (J.P.B.); mlujanmendezbauer@gmail.com (L.M.-B.); fagnerkiratzc@yahoo.com.br (F.K.); luisafer619@gmail.com (L.F.A.-A.); 5 Research Department, School of Dentistry, Universidad Francisco Marroquín (UFM), 6th Street 7-11 Zone 10, Guatemala City 01010, Guatemala; 6 Department of Restorative Dentistry, State University of Ponta Grossa (UEPG), Ponta Grossa, Paraná 840030-900, Brazil; pvfarago@gmail.com (P.V.F.); aloguercio@hotmail.com (A.D.L.); cesararrais@yahoo.com.br (C.A.G.A.); 7 Department of Dentistry, Cardenal Herrera-CEU University, CEU Universities, C/Santiago Ramón y Cajal, s/n., Alfara del Patriarca, 46115 Valencia, Spain

**Keywords:** universal adhesive system, flavonoids, microtensile bond strength, nanoindentation, nanoleakage

## Abstract

The aim of the present study was to evaluate the influence of several experimental pretreatment crosslinker solutions on the resin polymer–dentine interface created using a representative universal adhesive system, by means of microtensile bond strength testing (μTBS), nanomechanical properties and ultramorphology confocal laser scanning microscopy (CLSM). Five experimental solutions containing different flavonoids were applied as dentine pretreatment after acid etching. A control pretreatment group containing no flavonoid was also employed. A representative modern universal adhesive was then applied, followed by a 3 mm thick composite built up. Specimens were sectioned into sticks and submitted to a μTBS test or nanoindentation analysis along the interface (24 h or 25,000 thermocycles). The ultramorphology of the polymer–resin interface was also evaluated using CLSM. The results were analyzed using two-way ANOVA and Bonferroni’s post hoc test (α = 0.05). All flavonoids improved short- and long-term μTBS values (*p* < 0.01), while only some specific such solutions improved the nanomechanical properties (*p* < 0.05) and preserved the structural morphology of the interface after aging. Pretreatment of acid-etched dentine using specific flavonoid-containing solutions may be a promising approach to improve both the nanomechanical properties and the durability of modern universal adhesive systems.

## 1. Introduction

The bonding interface created by “simplified” adhesive systems is still considered the “Achilles′ heel” of resin-based restorations [1], since it is usually susceptible to severe degradation over time [2,3]. The reasons for such a situation can be attributed to a synergic hydrolytic degradation of the polymer matrix within the hybrid layer (HL) [4] and the enzymatic degradation of poorly infiltrated demineralized dentine collagen fibrils through activated host metalloproteinases (MMPs) [5] and cysteine-cathepsins (CTs) [6]. This is why substances capable of contrasting the proteolytic activity of activated MMPs and CTs, as well as the chemical hydrolytic degradation process of the polymer matrix, have been extensively evaluated in vitro and in vivo [7,8].

Exogenous crosslinkers such as flavonoids have demonstrated the ability to reduce the degradation of the hybrid layer [9]. Flavonoids are a group of natural substances with various polyphenolic structures. The major subclasses of flavonoids (anthocyanidins), flavan-3-ols, flavonols, flavanones, flavones, and isoflavones, are the most prevalent in the human diet. In general, these compounds show potent antioxidant, vasoactive, and antibacterial properties [10,11,12]. For instance, when oligomeric proanthocyanidins (PROs) were added to experimental HEMA-free adhesives, it was possible to modify the permeability of the dentine substrate (e.g., hydraulic conductance) and preserve the integrity of the HL over time [13].

Furthermore, the inclusion of specific flavanols, such as quercetin (QUE), improved the bonding performance, as well as the antibacterial properties of adhesive systems [14]. Likewise, other flavonoids, such as the flavanone hesperidin (HES), performed even better than the well-known PRO in reducing the degradation of the polymer-dentine interface [15,16]. Conversely, further flavonoids with similar chemical structures, such as rutin (RUT) and naringin (NAR), have not yet been thoroughly investigated.

Previous studies on the influence of polyphenolic compounds in dentine only compared the effect of such substances in aqueous solutions [17,18,19]. However, most flavonoid aglycones are insoluble or slightly soluble in water; this results in an aqueous suspension in which sedimentation is often encountered. Although, according to the Flory-Huggins theory, this solution may also affect the volume of the polymer matrix by means of swelling or plasticizing effects [20,21], it has been advocated that those flavonoids having more affinity with water may be also more effective when applied in dentine. Indeed, a study published by Fang et al. [22] demonstrated the importance of using ethanol as a vehicle when applying PRO to dentine; this improved the bonding performance of some specific adhesive systems compared to the group where only water was used as a vehicle. This aspect may be crucial when analyzing the effect of flavonoids as the best formulation can be obtained for each flavonoid based on its chemical and physical properties to interact with collagen and provide the maximum protective effect.

A recent study evaluated the impact of different experimental pretreatment solutions containing flavonoids on caries-affected dentine (CAD), showing promising results in preserving the HL [23]. In this latter study, each specific characteristic of the flavonoids was taken into account in order to create the most appropriate formulation to allow hydrophobic molecules to be available in a water–ethanol solution in caries-affected dentine. Considering the effect of flavonoids in a critical environment, the flavonoids′ impact in sound dentine becomes a relevant issue to be investigated in adhesive dentistry.

Thus, the aim of this study was to evaluate the influence of several experimental dentine pretreatment solutions based on HES, QUE, NAR, RUT, or PRO at a critical micelle concentration (CMC) applied prior to bonding procedures using a representative universal adhesive system applied in etch-and-rinse mode. Dentine microtensile bond strength (μTBS), as well as on nanoindentation (NH: hardness; E: modulus of elasticity), and confocal ultramorphology analysis of the resin polymer–dentine interface, were investigated either after 24 h or after 25,000 thermal cycles. The tested hypotheses were that the experimental crosslinking solutions applied prior to the bonding procedure to sound dentine (1) would improve µTBS values, (2) increase NH and E values along with the polymer–dentine interface, and (3) preserve the integrity of the HL.

## 2. Materials and Methods

**Figure 1:**
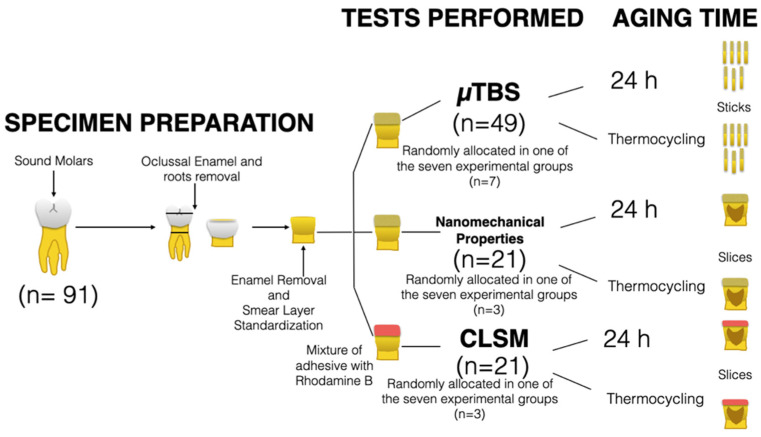
Graphical representation of the overall experimental design used in this study.

A general description of the experimental design can be seen in Figure 1.

### 2.1. Formulation of the Experimental Pretreatment Solutions

Five experimental water-ethanol solutions were prepared using a standard concentration (6.5%) of each of the following flavonoids: hesperidin (HES); quercetin (QUE); naringin (NAR); rutin (RUT) (Sigma Aldrich, St. Louis, MI, USA); grape seed (extraction from *Vitis vinifera*, high in proanthocyanidins) (PRO) (purity 95%) (Active Pharmaceutica, Palhoça, SC, Brazil). The purity, solubility index, hydrophobicity index, and the critical micelle concentration (CMC) of each flavonoid were kept in consideration for the formulation of the experimental pretreatment solutions used in this study (Table 1).

A specific composition for each flavonoid was obtained to allow the maximum availability of the active principle, but without jeopardizing their overall properties (Table 2) [22]. A placebo solution (PLA) containing only the vehicle used in the solution was obtained to quantify the effects of the active principles. A control group (CON) with the adhesive applied using none of the previous pretreatment solutions was also included in the study.

### 2.2. Preparation of Specimens and Application of the Adhesive System

Ninety-one caries-free extracted human third molars collected from patients (age: 18–35) were used. The teeth were collected under an approved protocol (Ethics Committee approval protocol number 41.2017) and after informed consent was obtained from each patient. Teeth were deposited in distilled water for no longer than three months. In order to exposed a flat mid-dentine surface, the occlusal enamel was abraded using 180-grit SiC abrasive paper. The dentine surface was then polished using 600-grit SiC paper to obtain a standardized and clinically relevant smear layer.

The dentine surfaces of each specimen were acid-etched with 37% phosphoric acid for 15 s (Ultra-Etch, Ultradent Products Inc., South Jordan, UT, USA), immediately rinsed with distilled water for 15 s, and finally dried using a tiny absorbent paper. Subsequently, the experimental solutions were applied through a micro-brush for 1 min to rewet the surface. They were subsequently blow-dried and the remining dentine moisture was standardized using absorbent paper in order to leave a slightly wet surface. A representative modern universal adhesive system (Scotchbond Universal Adhesive; 3M ESPE, Oral Care, MN, USA) was used as per the manufacturer’s instructions and light-cured for 10 s using an LED light-curing unit (VALO, Ultradent Products Inc., South Jordan, UT, USA). The irradiation output (>1000 mW/cm^2^) of the curing system was continuously checked using a radiometer (Bluephase meter II, Ivoclar Vivadent, Schaan, Liechtenstein). Subsequent to the bonding procedures, a buildup was constructed in three 1 mm thick increments using a light-curing resin composite (Opallis, FGM Prod. Odont. Ltd.a, Joinville, SC, Brazil); each composite layer was light-cured for 40 s (VALO, Ultradent Products Inc.).

Specimens were left for 24 h in distilled water at 37 °C. For the NH test, 21 restored teeth were longitudinally sectioned in the mesio-distal direction and across the composite—dentine interface using a diamond-embedded saw mounted on a cutting machine (IsoMet 1000, Buehler, Lake Bluff, USA), under continuous water cooling (300 rpm) to obtain resin–dentine slices approximately 1.2 mm thick (*n* = 3). For the μTBS analysis, 49 teeth were longitudinally cut in both “x” and “y” axial directions, across the composite–dentine interface in order to obtain resin–dentine sticks with a cross-sectional area of approximately 0.8 mm^2^. This latter was carefully measured using a digital caliper (0.01 mm) (Absolute Digimatic, Mitutoyo, Tokyo, Japan) for subsequent calculation of the μTBS values in MPa. Half of the sticks were evaluated (μTBS) after 24 h, while the remaining sticks were evaluated after aging (25,000 cycles; dwell time 30 s—5 °C to 55 °C) in a thermocycling machine (OMC 300TS, Odeme Dental Research, Joaçaba, SC, Brazil) [22].

### 2.3. Evaluation of Microtensile Bond Strength (µTBS)

Each resin–dentine stick was positioned onto a microtensile jig and glued using cyanoacrylate resin (IC-Gel, bSi Inc., Atascadero, CA, USA). These specimens were submitted to tensile force through a universal testing machine (Kratos, São Paulo, SP, Brazil) at 0.5 mm/min. Subsequent to the bonding fracture, each specimen was analyzed using an optical microscope (SZH-131, Olympus, Tokyo, Japan) at 40× to evaluate the failure mode. The type of fracture was classified as cohesive in dentine (failure exclusively within cohesive dentine-CD); cohesive in resin (failure exclusively within the resin-CR); adhesive (failure at resin polymer-dentine interface-A), or mixed (failure at resin/dentine interface that included cohesive failure of the surrounding substrates, M).

### 2.4. Nanoindentation: Hardness and Modulus of Elasticity across the Interface

Further teeth (*n* = 21) were restored as previously described and longitudinally cut to obtain composite–dentine slices (*n* = 3 each tooth). They were immediately polished using SiC papers (1000- to 4000-grit) for 30 s under continuous irrigation and finally treated for 8 min in the ultrasonic bath containing distilled water. The slice specimens were glued to a support stub and submitted to the nanoindentation test (UNAT nanoindenter, Asmec, Dresden, Germany), using the Berkovich indenter (20 nm radius). The resin polymer–dentine interface was visualized through the optical microscope of the nanoindenter system. A total of 24 indentations were performed (6 on the “x” axis and 4 on the “y” axis) at 5000 nN and a function time of 10 s. The analysis started at the adhesive layer (AL) and moved toward the HL and then down to the dentine surface and to a depth of 20 µm. Each indentation was performed at a distance of 10 µm (±1 µm) along the “y” axis and 100 µm (±10 μm) along the “x” axis. The results generated during the indentation test were analyzed to calculate the hardness (NH) and modulus of elasticity (E) at the polymer–dentine interface. This assessment was performed either after 24 h or after 25,000 thermocycles.

### 2.5. Confocal Laser Scanning Microscopy Analysis of the Adhesive Interface

The universal adhesive system used for this part of the study was mixed with 0.15 wt% Rhodamine B (83689-1G; Sigma-Aldrich, Munich, Germany) and applied subsequent to pretreatment of dentine with the experimental solutions, according to the bonding procedures explained above. Further teeth (*n* = 3 for each group) were prepared and stored in distilled water at 37 °C for 24 h. They were longitudinally sectioned across the bonded interface to obtain composite–dentine slices with a thickness of approximately 1.2 mm, as previously described. The thickness of each slice or cross-sectional area of each stick was measured with a digital caliper (Absolute Digimatic, Mitutoyo, Tokyo, Japan). Each slice was then immersed in 0.1 wt% sodium fluorescein water solution (46960-25G-F; Sigma–Aldrich, Munich, Germany) for four hours, as described in previous studies [23]. Subsequently, the specimens were polished using 1000-, 1500-, 2000-, and 2500-grit SiC papers and immediately treated in an ultrasonic bath with distilled water for 5 min. The specimens were then air-dried and stored in mineral oil. The resin polymer-dentine interfaces were analyzed with a confocal laser scanning microscope (CLSM: Leica TCLS, Leica, Heidelberg, Germany) using a 63X oil immersion lens (1.4 numerical aperture). Emission fluorescence was recorded at 512–538 nm (Fluorescein) and 585–650 nm (Rhodamine B). Ten z-stack (10 µm) images from each slice were randomly recorded. Leica LAS X software (Leica, Heidelberg, Germany) was used to analyze and reconstruct the obtained images into single projections.

### 2.6. Statistical Analysis

The results were initially assessed using the Shapiro-Wilk test to ascertain whether the data had a normal distribution. Moreover, Levene’s test was used to check the equality of variances and determine if the assumption of equal variances was valid. After confirming the normality of the data distribution and the equality of the variances, μTBS (MPa), nanohardness (Gpa), and modulus of elasticity (E), the results were subjected to two-way repeated-measures ANOVA, followed by Bonferroni’s post hoc test for pairwise comparisons (α = 0.05). The frequencies of the failure patterns were evaluated using a Pearson chi-squared test (α = 0.05) Post hoc power analysis was performed to analyze the data using commercial statistical software (Statistics 19, SPSS Inc, IBM Company, Armonk, NY, USA).

## 3. Results

The study had adequate statistical power for both factors (over 95%; α = 0.05) for µTBS, H, and E data

### 3.1. Microtensile Bond Strength Testing and Failure Mode Analysis

**Figure 2:**
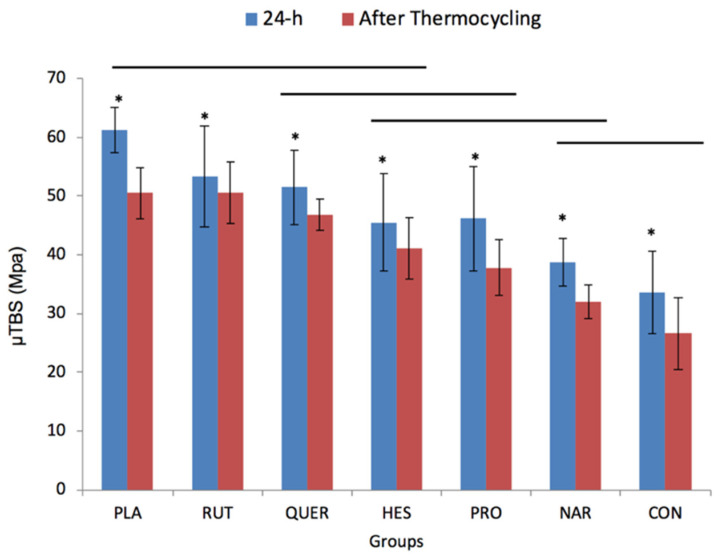
Bar graph showing mean values and standard deviation (SD) of the µTBS values (Mpa) obtained in the experimental groups at 24 h (blue) and after 25,000 cycles of thermocycling (red). Bars connected by horizontal black lines are not significantly different for both intervals (pre-set alpha of 5%). Asterisks indicate significant differences between intervals (pre-set α = 0.05).

The µTBS values (mean and standard deviation) are presented in Figure 2. Except for the NAR group, all the other experimental groups showed significantly higher µTBS values than the CON group (*p* < 0.01). The tested experimental solutions containing RUT and QUE had the highest µTBS values at both intervals (*p* < 0.001). Conversely, specimens in HES, PRO, and NAR groups exhibited significantly lower values than those in the PLA and RUT groups (*p* < 0.05). The specimens in the CON group showed the lowest µTBS values at both intervals (*p* < 0.001), with no significant difference compared to the NAR group. All experimental groups presented a significant drop in µTBS values after thermocycling (*p* < 0.01).

**Figure 3:**
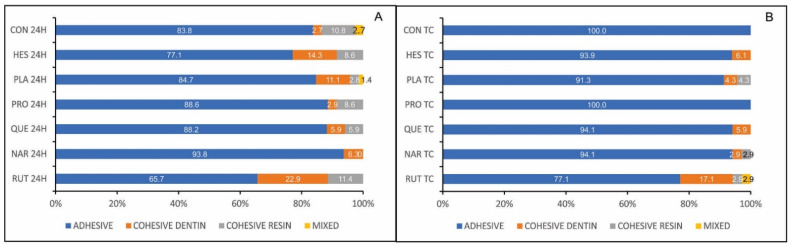
Distribution of failure pattern in the experimental groups at 24 h (**A**) and after thermocycling (**B**).

The Pearson χ^2^ test showed that the failure pattern was significantly influenced by treatment and experimental time (for treatment, χ^2^ = 33.948, *p* = 0.013; for experimental time, χ^2^ = 13.282, *p* = 0.004). The adhesive failure pattern was predominantly observed in all experimental groups. The specimens in the RUT group exhibited the highest percentage of cohesive failures within dentine among all groups, regardless of the experimental time (24 h or after thermocycling). After thermocycling, the percentage of cohesive failures decreased in the experimental groups (Figure 3B), exhibiting a prevalent failure in adhesive mode. Such a change in failure pattern over time was also observed in the CON, HES, and PLA groups (Figure 3).

### 3.2. Nanoindentation: Hardness and Modulus of Elasticity across the Interface

The mean NH and E values obtained in the adhesive and hybrid layers are displayed in Table 3, while the values at 10 and 20 µm along the dentine surface are shown in Table 4. At the adhesive layer, HES and NAR exhibited the highest NH and E values (*p* < 0.001), while HES and QUE improved the nanomechanical properties (*p* < 0.001) of the hybrid layer. The experimental flavonoid-containing solutions induced no significant change (*p* > 0.05) compared to the CON group. The specimens in the PLA group showed the lowest NH and E values both in the adhesive and hybrid layer (*p* < 0.05). A significant drop in NH and E values after TC was noted at the HL in all experimental groups. However, the specimens in the NAR and HES groups exhibited a significant drop in NH and E values after TC both at the adhesive and hybrid layer (*p* < 0.001). After thermocycling, no significant differences (*p* > 0.05) were observed between the groups in NH and E values at the adhesive and hybrid layers.

No significant interaction was detected between factors in the two-way repeated-measures ANOVA for the results obtained in dentine at 10 or 20 µm. Hence, the final comparison was performed between the total average (mean) of the results attained in each group at 24 h or after 25,000 thermocycles. No significant differences in NH and E values were found between the experimental groups in dentine at 10 and 20 µm (Table 4). The specimens in the HES and RUT groups presented the highest mean values in both NH and E tests, while those in the NAR group obtained the lowest values (*p* < 0.01). At a 20 µm depth, the lowest NH and E values were observed in the specimens of the PLA and NAR groups, respectively (Table 4). The remaining groups showed no significant differences at 10 or 20 µm. A significant decrease in NH and E values was observed after TC in all experimental groups (*p* < 0.001).

### 3.3. Confocal Laser Scanning Microscopy Analysis of the Adhesive Interface

**Figure 4:**
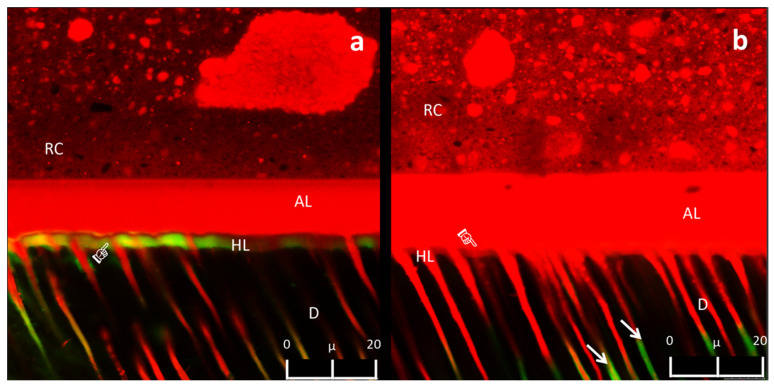
(**a**) Representative CLSM image (63X, Zoom 2X, depth: 10 μm) of the fluorescein infiltration in specimens of CON, QUE, and HES groups at 24 h. The presence of fluorescein was noted in some regions within the HL (pointer). (**b**) Representative CLSM image (63X, Zoom 2X, depth: 10 μm) of the nanoleakage in the experimental RUT, PLA, PRO, and NAR groups after 24 h. No leakage was noted within the HL in these groups (pointer). Some fluorescein was noted within the dentine tubules (white arrows). Resin composite (RC), adhesive layer (AL), hybrid layer (HL), dentine (D).

**Figure 5:**
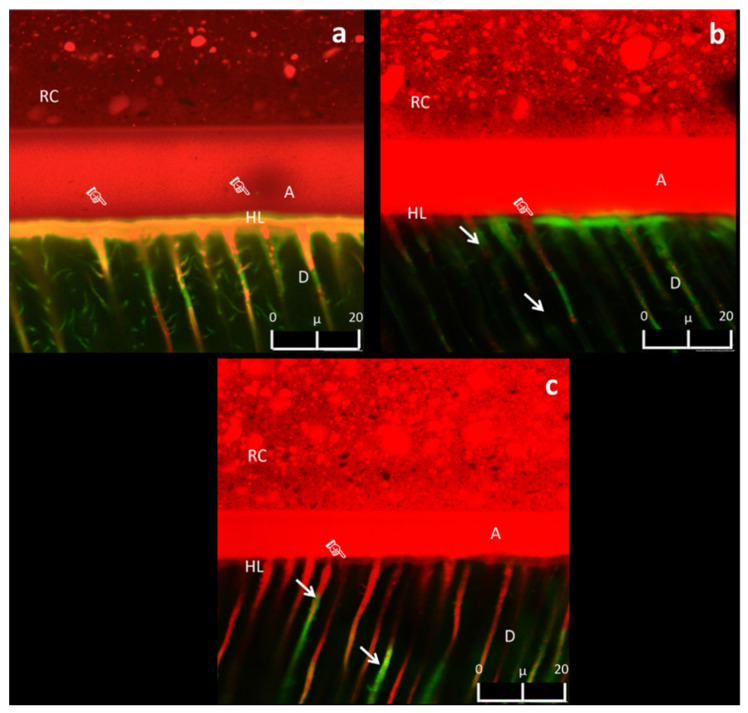
(**a**) Representative CLSM image (63X, Zoom 2X, depth: 10 μm) of the fluorescein infiltration observed in the specimens in the CON group after thermocycling. Orange shaded hybrid layer as a result of the mixture between fluorescein and rhodamine B was noted (pointer). (**b**) Representative CLSM image (63X, Zoom 2X, depth: 10 μm) of the nanoleakage in the experimental QUE and NAR groups after thermocycling. Infiltration of fluorescein was noted at the bottom of the hybrid layer (pointer), as well as the presence of fluorescein surrounding resin tag ends (white arrow). (**c**) Representative CLSM image (63X, Zoom 2X, depth: 10 μm) of the nanoleakage in the experimental RUT, PRO, and PLA groups after thermocycling. Infiltration of fluorescein at the bottom of the hybrid layer was noted (pointer). Presence of fluorescein surrounding the resin tag ends (white arrow). Resin composite (RC), adhesive layer (AL), hybrid layer (HL), dentine (D).

The CLSM analysis showed intense fluorescein infiltration at the bottom of the HL of the specimens in CON, QUE, and HES groups at 24 h (Figure 4a). Conversely, such a diffusion of fluorescein was not observed within the HL of the specimens in PLA, PRO, RUT, and NAR groups at 24 h (Figure 4b), but the fluorescein only penetrated the dentine tubules up to 20 µm, far away from the HL (Figure 4b). After thermocycling, the CON group exhibited intense areas of fluorescein infiltration within the HL (Figure 5a). On the other hand, the specimens in groups QUE and NAR maintained a similar pattern of fluorescein infiltration (Figure 5b) compared to the specimens at 24 h. In contrast, a slight diffusion of fluorescein within the HL was observed in the specimens in the RUT, PRO, and PLA groups (Figure 5c). In these latter groups, the HL remained apparently stable after thermocycling, with almost no presence of gaps or fluorescein infiltration at the bottom of the hybrid layer (Figure 5c).

## 4. Discussion

**Figure 6:**
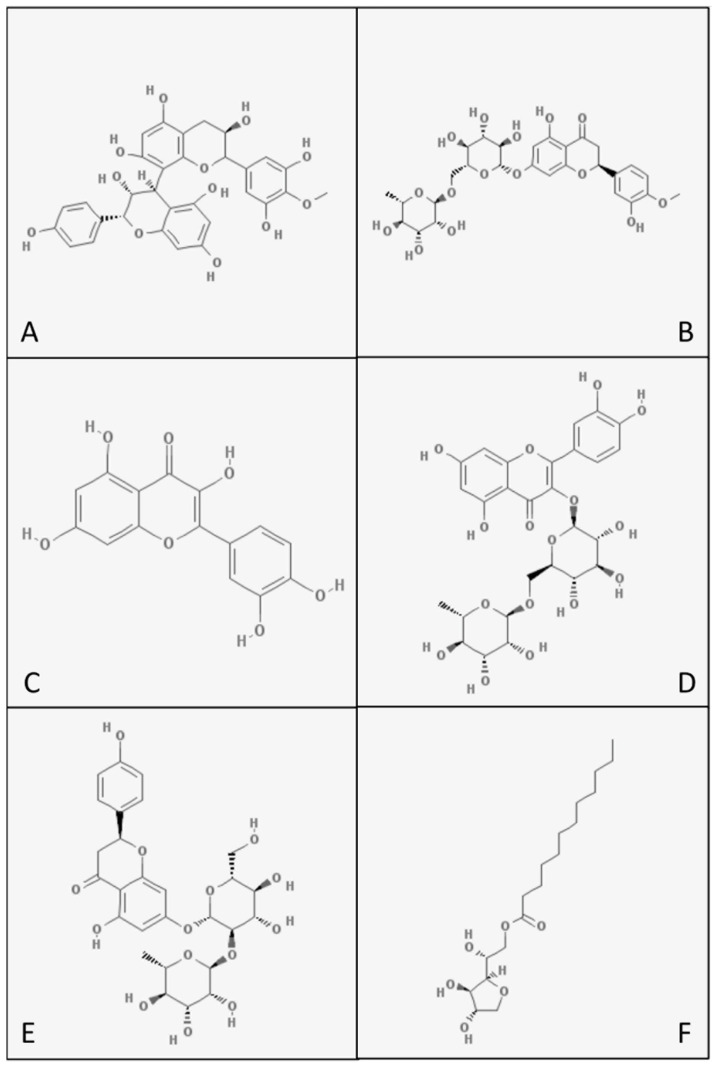
Flavonoid and surfactant molecules: proanthocyanidin (**A**), hesperidin (**B**), quercetin (**C**), rutin (**D**), naringin (**E**), SPAN 20 (**F**).

The µTBS values of the specimens pretreated with some specific flavonoids were higher than the values of the specimens in the CON group. Thus, the first hypothesis must be partially accepted. Although the current results corroborate previous studies, [22,23,24,25], the effectiveness of the tested flavonoids varied between the tested groups; the chemical characteristics of each molecule seem to influence the final results. The mechanisms involved in the interaction between the flavonoids and dentine have been shown to be correlated to the potential formation of covalent and hydrogen bonds [26,27]. Such an interaction may be dependent to the number of reactive phenolic groups, as well as to the amount and size of the reactive groups available in each molecule, which can react with hydroxyl, carboxyl, amine, or amide groups in the collagen fibrils [28,29,30] (Figure 6). However, based on the unexpected failure of PRO when used as a primer solution [31], or when included in the composition of an adhesive system [32], the effectiveness of crosslinking molecules seems to be more complex in a clinical setting, due to the fact that several different substrates may interact and react simultaneously. In other words, the composition and acidity of different types of dentine substrates (i.e., caries or sound dentine), as well as the composition of the adhesive systems, may also play an essential role in the mechanisms of interaction between flavonoids and dentine.

In this study, the specimens in the RUT group showed the highest µTBS values between the tested flavonoids. Even though this molecule does not have the largest amount of potential phenolic sites to bind to collagen, the current results may be explained by some aspects related to the molecular structure: (1) the critical micelle concentration (CMC) in the solution; (2) the index of solubility of the molecule, which may have influenced its dissolution in the selected vehicle; (3) the number and type of reactive molecule sites; (4) the potential chemical interaction with the adhesive system; (5) the small molecular size, which may be able to diffuse within the collagen network, leading to an increase in the nanomechanical properties of dentine (Table 4). The benefits of using flavonoids with low molecular weight have been previously reported by Liu et al. [33].

Conversely, the pretreatment solution containing NAR was not as effective as the pretreatment containing other flavonoids; the µTBS values were not significantly different compared to the CON group. Despite its molecular weight and molecular similarity to the other flavonoids, this molecule has some disadvantages that may have compromised its performance. For instance, it presents few reactive phenolic sites, high molecular mass, and low solubility in water; it is well known that the hydrophilicity of flavonoids increases according to the number of phenolic sites (Table 2). In each glycoside flavone NAR molecule, there are only two phenolic groups available. Its heterocyclic ring has only one phenolic site, while the other is located on the terminal phenolic group. With such a limited number of free phenolic sites, NAR may dissolve better in lipid solutions or in other solutions at high temperatures. Moreover, NAR might be more reactive when dissolved in solvents such as ethanol [34]. However, the formation of larger chains in neutral and/or alkaline environments might compromise its protective performance when applied in sound dentine [35].

The results obtained in the current study are in discordance with those presented in a previous investigation, which showed a beneficial effect of NAR in caries-affected dentine [23]. Moreover, the same study showed that the buffer effect of caries-affected dentine was reduced due to the lower mineral content of that substrate. Such a lower pH in CAD can make organic compounds prone to changes in the last electron orbits of their molecular structure, producing different chemical interactions of flavonoids with the collagen fibrils [23]. Furthermore, an acidic pH allows better diffusion of organic molecules; a phenomenon called lixiviation. In this sense, the substrate′s pH where the flavonoid is applied may play an essential role in activating different molecular sites of organic compounds.

The NH and E values in the adhesive and hybrid layers of the specimens in the experimental groups were higher than those observed in the CON group, therefore, in this case, the second hypothesis must also be partially accepted. The tested flavonoids effected both the dentine substrate and the resin polymer matrix of the adhesive system used in this study. Some studies demonstrated that compounds containing catechol-type molecules in their formulations [36,37,38,39] which could chemically interact with the acrylate groups of dental polymers [38,40]. Consequently, the copolymerization of flavonoids with the bonding agent may have resulted in ester-type chemical bonds that improved the mechanical properties of the adhesive layer [38,40]. Regarding to the effect attained in the hybrid layer, those results could not be exclusively attributed to the ability of flavonoids to co-polymerize with the methacrylate groups in the adhesive layer, but also other factors, such as (1) the therapeutic effect in dentine of crosslinking molecules; the crosslinking formation within collagen as a result of the bio-modification of the mechanical properties. This effect was seen mainly within and underneath the hybrid layer, on the exposed and unprotected collagen fibrils. (2) The changes in dentine humidity as a result of the high-density crosslinking of flavonoids within collagen; this may have increased the monomer conversion of the adhesive.

Conversely, a significant drop in the nanomechanical properties at the polymer–dentine interface was observed in some of the experimental groups after thermocycling. We might attribute such a result to the aging method used in this study. Indeed, many organic compounds, including the tested flavonoids, can be susceptible to oxidation due to excessive temperature stress over time [41]. Despite the transitional effect that some flavonoids exhibited in the hybrid layer and dentine, the initial therapeutic effect must not be ignored, as in a clinical scenario, adhesive layers are surrounded by resin composites or other restorative materials, which may be able to reduce the “deleterious” effect of the temperature shock along the interface, minimizing the thermal oxidative effect in flavonoids [42]. Moreover, we should also consider that the improved mechanical properties of hybrid layers may be a result of better polymerization of the bonding agent within the demineralized dentine. This aspect may imply that a higher degree of conversion can be achieved in such a scenario. However, further investigation is required to better address this scenario.

When analyzing the CLSM results obtained in this study, it was observed that the specimens of the experimental groups presented less fluorescein infiltration at the bottom of the adhesive layer and within the HL compared to those of the CON group. In this case, the third hypothesis must be accepted. A recent study demonstrated the ability of flavonoids to modify the relative humidity and permeability of dentine [13]. This effect was attributed to the high density of crosslinks formed by oligomeric proanthocyanidins in dentine, which reduced the surface hydrophilicity of the dentine [27], as well as the intrinsic hydrodynamics of the dentinal tubules [43]. These factors may have favored the interaction of the flavonoids with the dentine collagen, which caused water displacement and better infiltration of the resin monomers into the acid-etched dentine. Furthermore, flavonoids in synergism with the solvents employed in this study may have improved the displacement of proteoglycans within the collagen network, which are responsible for keeping the dentine wet [13]. Thus, the infiltration of resin monomers may have been facilitated in such a “less humid” dentine surface. Indeed, the results of this study showed a lower level of nanoleakage within the HL of the specimens in the experimental groups, even after thermocycling challenge (Figure 4 and Figure 5).

A significant decrease in µTBS values was seen after thermocycling in all experimental groups. As previously stated, it is important to consider that organic molecules, such as flavonoids, can undergo oxidation at high temperatures [40]. Therefore, this may be one of the main reasons why crosslinking properties were impaired in this study after thermocycle aging. This effect may be also confirmed by the decrease in the nanomechanical values observed in the HL in most of the experimental groups after thermocycling. Conversely, the nanomechanical properties of the polymer–dentine interface of the specimens in the CON and PLA groups (with no flavonoids) were not affected by thermocycling. This latter result may support the hypothesis that high temperatures jeopardize the “therapeutic” effects of flavonoids.

Interestingly, the specimens in the PLA group presented the highest µTBS values after 24 h between all the tested experimental groups. The PLA pretreatment solution was formulated using the same vehicle used in the experimental solutions (e.g., ethanol and a surfactant, SPAN 20). The experimental solutions were designed to use the smallest ethanol volume to avoid further changes on the dentine surface. Once PLA was applied without any flavonoid, the amphiphilic molecules in that pretreatment solution were fully available, so they may have increased the surface energy of dentine, and improved the infiltration of the adhesive resin monomers into the acid-etched dentine collagen network [44]. It is also possible that ethanol might have influenced the azeotropic properties of the adhesive system during its application [45], enhancing the displacement and evaporation of water from dentine. We hypothesize that these factors are the main ones responsible for obtaining no significant changes in NH and E values in some specific experimental groups rather than in the specimens of the CON group. The high µTBS values observed in the PLA group may be related to an improved quality of adhesive infiltration within the demineralized dentine, as seen in the CLSM analysis (Figure 4b). Indeed, the overall substrate humidity may have been influenced by the surfactant used within the composition of the experimental solutions. In fact, because of the amphiphilic properties of SPAN 20, there must have been an improvement of the chemical interactions with collagen related to the presence of both hydrophilic and hydrophobic active sites in this molecule [46]. Thus, in view of this latter aspect, one might affirm that the increase in the µTBS values in all experimental groups is attributable exclusively to the effect of the vehicle (e.g., ethanol) added to the experimental solutions to achieve the critical micelle concentration. However, it should be considered that there was a drop in µTBS after thermocycling in most of the experimental groups, that ranged from 5.3% to approximately 9.8%, while the µTBS value drop in the PLA group was about 17.6%. Such a decrease in µTBS values in the experimental groups in comparison to those observed in the specimens of the PLA group may be attributed to the crosslinking properties of the flavonoids discussed above; their effects in collagen have been widely demonstrated in many biomedical areas [47,48,49,50,51]. Indeed, such “therapeutic” effects may have prevented the µTBS values from dropping as much as the drop observed in the specimens of the PLA and CON groups. This may be interpreted as an indirect evaluation of flavonoids′ substantivity over time. Nevertheless, the synergic influence of the vehicle with the effects provided by the flavonoids on the increase of the µTBS value in some experimental groups should not be disregarded.

In contrast to some previous in vitro studies that evaluated the effects of flavonoids in demineralized dentine [17,18,19], the composition of the experimental pretreatment solutions employed in the current study was based on the critical micelle concentration of the flavonoids in order to allow the maximum interaction with the dentine collagen. Each flavonoid molecule has a different water solubility index, which results in more dissolution in a specific liquid; this concept may have had a determinant role in the therapeutic effect of flavonoids used in this study [52]. It is important to consider that only one representative universal adhesive system was evaluated in the present study. It was decided to use such a bonding strategy to simulate a challenging clinical scenario. Indeed, when dentine is etched with phosphoric acid, collagen fibrils are exposed and they become more susceptible to degradation [1]. Moreover, such a bonding approach may allow the diffusion of crosslinking molecules into the demineralized dentine collagen network. Therefore, the current results should not be translated to a clinical scenario where the adhesive system is used in self-etching mode. However, despite the improved µTBS values and nanomechanical properties, the dentine surface treatment proposed in this study requires that dentists perform an additional step during bonding procedures, which may make this approach more technique sensitive and time consuming for daily practice. The variability in the therapeutic effect of flavonoids and the influence that the environment can produce in these substances are essential aspects to consider for the development of new materials. Further investigation is necessary to better understand the potential clinical benefits of such flavonoid-containing materials.

## 5. Conclusions

Within the limitations of this study, we may affirm that the use of flavonoids may lead to an improvement of the bonding performance of simplified universal adhesives when applied in etch-and-rinse mode on sound dentine, without jeopardizing their nanomechanical properties, even after a severe aging challenge such as thermocycling. Pretreatments with flavonoid-containing solutions might be a therapeutic alternative material to reduce the permeability of hybrid layers, and improve the longevity of the resin polymer-dentine interface over time.

## Figures and Tables

**Table 1:** Composition of the water-ethanol flavonoid solutions.

Component	Compound	Quantity %
Active Compound	Flavonoid	6.5% mass
Vehicle (Pure Ethanol)	Pure Ethanol	30% (3 mL)
Surfactant (Polysorbate 20)	SPAN 20	1% (0.1 g)
Aqueous Medium	Distilled Water	QS 10 mL

**Table 2:** Physical and chemical properties of the molecules used in this study.

Substance	Molecular Mass	Number of Hydroxyphenyl Radicals	Number of Alcoholic Radicals	Number of Mols (6.5% Mass)	Solubility in Water
Hesperidin	610.56 g/mol	2	6	1.06 mm	0.02 mg/mL
Naringin	580.53 g/mol	2	6	1.12 mm	1 mg/mL at 40 °C
Proanthocianydin	595.55 g/mol	7 *	2 *	1.09 mm *	0.130 mg/mL *
Quercetin	302.24 g/mol	5	-	2.15 mm	0.06 mg/ml
Rutin	610.52 g/mol	4	6	1.06 mm	0.125 mg/ml

* Expected chemical and physical properties of the proanthocianydin repeating unit (mer), which may vary according to the number of repeating units (mers) in the final molecule (oligomer or polymer), reducing solubility and increasing the molecular mass according to the size of the final chain.

**Table 3:** Mean of the nanomechanical properties nanohardness (NH) and modulus of elasticity (E) values (standard deviation) in GPa of the adhesive and hybrid layers created using bonding agent with flavonoids at 24 h and after thermocycling.

Indentation Site	Flavonoid	NH 24 h	NH Thermocycling	E 24 h	E Thermocycling
Adhesive Layer	PLA	0.265 (0.051) ^Ac^	0.256 (0.018) ^Aa^	6.55 (0.86) ^Ac^	5.63 (0.89) ^Aa^
	RUT	0.282 (0.002) ^Abc^	0.249 (0.017) ^Aa^	7.10 (0.88) ^Ac^	6.29 (0.56) ^Aa^
	QUE	0.274 (0.022) ^Ac^	0.250 (0.011) ^Aa^	7.14 (1.44) ^Ac^	6.16 (0.31) ^Aa^
	HES	0.444 (0.076) ^Aa^	0.285 (0.039) ^Ba^	12.90 (2.77) ^Aab^	7.40 (0.76) ^Ba^
	PRO	0.290 (0.051) ^Aabc^	0.244 (0.024) ^Aa^	7.81 (1.59) ^Abc^	7.20 (0.86) ^Aa^
	NAR	0.440 (0.061) ^Aab^	0.306 (0.018) ^Ba^	13.60 (1.82) ^Aa^	8.89 (3.27) ^Ba^
	CON	0.275 (0.07) ^Ac^	0.286 (0.040) ^Aa^	7.48 (1.96) ^Ac^	7.66 (0.86) ^Aa^
Hybrid Layer	PLA	0.330 (0.04) ^Ad^	0.350 (0.060) ^Aa^	10.79 (0.88) ^Ad^	10.86 (1.19) ^Aa^
	RUT	0.460 (0.04) ^Abcd^	0.310 (0.010) ^Ba^	13.64 (0.29) ^Acd^	9.58 (0.56) ^Ba^
	QUE	0.640 (0.11) ^Aab^	0.320 (0.010) ^Ba^	18.14 (3.28) ^Aab^	9.31 (0.42) ^Ba^
	HES	0.740 (0.01) ^Aa^	0.340 (0.010) ^Ba^	22.51 (0.18) ^Aa^	8.30 (2.10) ^Ba^
	PRO	0.520 (0.03) ^Abc^	0.380 (0.040) ^Ba^	14.90 (0.85) ^Abc^	12.36 (3.31) ^Aa^
	NAR	0.580 (0.07) ^Aab^	0.320 (0.090) ^Ba^	18.97 (0.77) ^Aa^	9.05 (2.57) ^Ba^
	CON	0.367 (0.08) ^Acd^	0.384 (0.030) ^Aa^	12.44 (0.61) ^Acd^	11.43 (0.98) ^Aa^

Means followed by same letter (upper case letters: within row; lower case letter: within column) are not significantly different (pre-set alpha: 0.05).

**Table 4:** Mean of the nano-mechanical properties nanohardness (NH) and modulus of elasticity (E) values (standard deviation) in GPa of the dentine layers (10 µm and 20 µm) at 24 h and after thermocycle aging.

10 μm Dentin			PLA	RUT	QUE	HES	PRO	NAR	CON	Average
	NH	24 h	0.649 (0.096)	0.767 (0.055)	0.757 (0.024)	0.788 (0.053)	0.698 (0.042)	0.637(0.078)	0.677 (0.141)	0.711 ^A^
		Thermocycling	0.574(0.055)	0.633 (0.105)	0.560 (0.047)	0.690 (0.072)	0.609 (0.067)	0.464 (0.028)	0.663 (0.042)	0.599 ^B^
		Average	0.612 ^ab^	0.700 ^a^	0.659 ^ab^	0.739 ^a^	0.654 ^ab^	0.550 ^b^	0.670 ^ab^	
	E	24 h	20.27 (1.22)	23.20 (0.32)	22.86 (1.12)	24.52 (0.70)	21.20 (0.87)	20.93 (1.02)	21.94 (0.99)	22.13 ^A^
		Thermocycling	16.44 (4.35)	18.16 (1.52)	17.47 (1.21)	20.25 (3.30)	19.06 (1.50)	13.24 (4.31)	20.52(0.67)	17.88 ^B^
		Average	18.35 ^ab^	20.67 ^ab^	20.16 ^ab^	22.38 ^a^	20.13 ^ab^	17.08 ^b^	21.23 ^ab^	
20 μm Dentin	NH	24 h	0.660 (0.074)	0.819 (0.079)	0.745 (0.028)	0.786 (0.053)	0.720 (0.054)	0.633 (0.10)	0.738 (0.069)	0.730 ^A^
		Thermocycling	0.578 (0.073)	0.762 (0.048)	0.680 (0.079)	0.743 (0.032)	0.712 (0.083)	0.547 (0.094)	0.685 (0.127)	0.670 ^B^
		Average	0.619 ^b^	0.791 ^a^	0.713 ^ab^	0.764 ^a^	0.716 ^ab^	0.590 ^b^	0.712 ^ab^	
	E	24 h	21.13 (1.55)	25.09 (0.86)	23.12 (1.15)	24.15 (0.40)	21.96 (0.16)	20.98 (0.68)	23.16 (0.19)	22.80 ^A^
		Thermocycling	19.61 (0.51)	21.832 (0.86)	21.9 (1.15)	22.85 (0.54)	21.75 (1.87)	18.74 (0.44)	21.17 (1.66)	21.13 ^B^
		Average	20.37 c	23.46 ^ab^	22.53 ^ab^	23.50 ^a^	21.86 ^abc^	19.86 ^c^	22.16 ^abc^	

Means followed by same letter (upper case letters: within column; lower case letter: within row) are not significantly different (pre-set alpha: 0.05).

## Data Availability

Not applicable.
